# *Vibrio furnissii*, an emerging pathogen causing acute gastroenteritis: a Case Report

**DOI:** 10.1099/jmmcr.0.005111

**Published:** 2017-09-12

**Authors:** Mamatha Ballal, Vignesh Shetty, Sohan Rodney Bangera, Mukhyaprana Prabhu, Shashikiran Umakanth

**Affiliations:** ^1^​ Enteric Diseases Division, Central Research Lab, Kasturba Medical College, Manipal University, Manipal, Karnataka, India; ^2^​ Department of Medicine, Kasturba Medical College, Manipal University, Manipal, Karnataka, India; ^3^​ Department of Medicine, Dr. TMA Pai Hospital, MelakaManipal Medical College, Manipal University, Udupi, Manipal, Karnataka, India

**Keywords:** *Vibrio furnissii*, Gastroenteritis, immunosuppressed, doxycycline

## Abstract

**Introduction.** *Vibrio furnissii* is a motile, Gram-negative, oxidase-positive, halophilic bacteria first defined in 1977. It is ubiquitously present in marine environments and is one of the 11 non-cholera *Vibrio* species pathogenic in humans, which can lead to human gastroenteritis and extra-intestinal manifestations.

**Case presentation.** A 73-year-old female patient was admitted to the hospital with acute gastroenteritis after consumption of seafood, which later by microbiological investigations was confirmed as *Vibrio furnissii*, a member of the family *Vibrionaceae*. The patient was treated with oral doxycycline and ciprofloxacin.

**Conclusion.** *V. furnissii*, an emerging pathogen known for quite some time as an aetiological agent responsible, for acute gastroenteritis cases yet to get more clinical attention. Descriptions of putative virulence factors of this pathogen are limited, and in-depth studies on the pathogenesis of *V. furnissii* need to be established.

## Abbreviations

MALDI-TOF, matrix-assisted laser desorption ionization time-of-flight; MS, mass spectrometry.

## Introduction

*Vibrio furnissii* is a motile, Gram-negative, oxidase-positive, halophilic bacteria first defined in 1977 and subsequently isolated from diarrhoeal and environmental sources [1]. *Vibrio furnissii* and *Vibrio fluvialis* are exceedingly alike in their phenotypic characteristics, however *V. furnissii* is differentiated from *V. fluvialis* by its production of gas from the fermentation of carbohydrates and also by the sequence differences in the genes *toxR* and *rpoB* [1]. It is one of the 11 non-cholera *Vibrio* species pathogenic in humans [2]. These ‘emerging *Vibrio* species’ include *V. furnissii*, a widespread, free-living, marine species that is associated with acute gastroenteritis [3]. *Vibrio furnissii* from human gastroenteritis is rarely reported and clinical characteristics of infections with this organism have not been well reported [4]. The disease typically occurs after ingesting contaminated raw or undercooked seafood or after contact with warm marine environments [2]. Here we describe a patient who developed gastroenteritis from *V. furnissii* and was successfully treated with oral antibiotics doxycycline and ciprofloxacin.

## Case report

A 73-year-old female patient was admitted to our hospital on 17 July 2016. She had been suffering from acute gastroenteritis for about 10 days with 2–3 episodes of loose stools per day.

There was no history of vomiting, abdominal pain or fever in the patient. She had consumed fried sea-fish before the onset of diarrhoea. The patient had type 2 diabetes mellitus for about 9 years. She also had a past history of hypothyroidism and osteoporosis. She had been previously treated for colonic cancer 16 years earlier and was on intermittent treatment for irritable bowel syndrome for 5 years. She had also undergone right hip replacement surgery about 2 years ago.

## Investigations

Stools passed were watery, not bloodstained, without melena and non-purulent. On examination, the patient’s vital signs were normal with respiratory rate 16 breaths per minute, blood pressure (BP) 130/70 mmHg, pulse rate 88 beats per minute. No fever and no signs of dehydration were seen. No abnormalities were detected in the cardiovascular, central nervous and respiratory system. Her abdomen was soft and non-tender. No organomegaly was found. Her potassium levels were low, and white blood cell count (8.5×10^3^ µl^−1^) was found to be normal. Initially the patient was started on empirical treatment with intravenous ceftriaxone, 2 g once daily. Subsequently, after the stool culture suggested *Vibrio furnissii,* oral doxycycline, 100 mg twice daily, was added. The patient continued to have 4–6 episodes of diarrhoea per day. Later, ceftriaxone was replaced by oral ciprofloxacin, 500 mg twice daily. Repeated stool culture sent after treatment was found to be negative for *Vibrio furnissii.* The patient improved symptomatically too and was discharged home.

## Diagnosis

The stool specimens from the above case were received at the microbiology laboratory attached to a tertiary care hospital (Kasturba Medical College Hospital, Manipal, India). Macroscopic observation of the stool samples revealed a watery stool with no blood and mucus in it. Occult blood was negative, microscopic examination showed moderate WBC and macrophages but no RBC/ova/cyst/trophozoites. The specimens were then inoculated into blood agar, MacConkey agar, selenite faeces broth, alkaline peptone water and hekton enteric agar as a standard laboratory protocol [5]. After overnight incubation at 37 °C, predominant beta-haemolytic colonies from blood agar obtained from alkaline peptone watersubculture were subjected to a battery of biochemical tests. The isolate was oxidase-positive, gave acid/acid with gas on triple sugar iron agar with no H_2_S, reduced nitrate to nitrite, utilized citrate as a sole source of carbon, did not produce indole, was methyl red-positive, Voges–Proskauer-negative and urease-negative, fermented d-glucose with gas, showed arginine dihydrolyase activity, but lysine and ornithine were not decarboxylated. The colonies were also confirmed as *Vibrio furnissii* by using matrix-assisted laser desorption/ionization time-of-flight mass spectrometry (MALDI-TOF MS). The repeat samples sent also yielded *Vibrio furnissii*. Antibiotic susceptibility testing of the isolates was done by the standard Kirby–Bauer disc diffusion method following Clinical and Laboratory Standards Institute (CLSI) guidelines. The isolate was found to be resistant (µg per disc) to ampicillin (10), and sensitive (µg per disc) to ceftriaxone (30), ciprofloxacin (5), chloramphenicol (30), trimethoprim/sulfamethoxazole (1.25/23.75), tetracycline (30) and gentamicin (30) (Table 1).

**Table 1.** Antimicrobial susceptibility results of *Vibrio furnissii*

**Antimicrobial**	***Vibrio furnissii***
Ampicillin	r
Ceftriaxone	s
Chloramphenicol	s
Ciprofloxacin	s
Gentamicin	s
Tetracycline	s
Trimethoprim/sulfamethoxazole	s

r, Resistant; s, susceptible.

## Treatment

According to the antimicrobial susceptibility report, the patient was treated with doxycycline, 100 mg orally given twice daily, with ciprofloxacin added later.

## Outcome and follow-up

After treatment the patient was symptomatically better, and no intestinal pathogens were reported from her repeat stool microbiological investigations.

## Discussion

*Vibrio furnissii* is ubiquitously present in aquatic marine environments [2] and in the intestines of healthy brown shrimp [6]. Infections caused by these vibrios are often associated with ingestion of contaminated seafood/exposure to coastal waters [2].

*V. furnissii* has also been associated with outbreak and sporadic cases of human gastroenteritis [1, 2, 4, 7, 8]. A case of *V. furnissii* bacteraemia with associated bilateral lower extremity lesions was also reported [9]. In this study, we found *V furnissii* causing gastroenteritis, which is the first case report from India, with only a few cases being reported from other countries.

Initially, *V. furnissii* was taxonomically assigned with *V. fluvialis* and named as aerogenic biogroup of *V. fluvialis*. Based on DNA relatedness and several biochemical tests, *V. furnissii* has been separated as a new species [9]. In the phylogenetic analysis with several housekeeping genes, *V. furnissii* and *V. fluvialis* have been linked as close species. The nucleotide comparison of 16S rRNA gene, *recA* and *toxR* sequences showed that *V. furnissii* and *V. fluvialis* had 100 % similarity. With the *gyrB* sequence, there was 93 % similarity shared by *Vibriao cholerae*, *Vibrio mimicus*, *V. furnissii* *and V. fluvialis* [10].

The relative pathogenicity of *V. furnissii* in most of these instances of human gastroenteritis was unclear, in that other pathogens may have contributed to the disease or that the individuals were asymptomatic at the time of stool collection. Some factors suggested as contributing to virulence in *V. furnissii* have been reported. Flagellum is a virulence factor in *Vibrio* as well as several bacteria [11]. The property of *V. furnissii* culture supernatants to lyse erythrocytes and their lethal effects on epithelial cells is a remarkable feature of pathogenesis [4]. In addition to the pathogenic factors of *Vibrio* such as proteases, haemagglutinins and other hydrolytic exoenzymes, haemolysin is one among them responsible for its pathogenesis [12]. The lipopolysaccharide component also plays an important role in the virulence by preventing the formation of the complement membrane attack complex thus precluding cell lysis [13].

In our case, the *V. furnissii* isolated was found to be resistant to ampicillin and sensitive to all other antibiotics like cotrimoxazole, gentamicin, chloramphenicol, tetracycline and ciprofloxacin. Other reports also reveal the same pattern of resistance to the antibiotics [7, 9]. No other family members of the patient who had consumed the same food were affected. The immunocompromised conditions of the patient such as age, diabetes mellitus and other illnesses could have resulted in her having acute gastroenteritis. A literature survey from the PubMed database shows no reports of *V. furnissii* being isolated from humans in India. Though the emerging pathogen *V. furnissii* has been known for quite some time, its clinical importance is not well documented in the literature. There are not many established in-depth studies done on the pathogenesis of this emerging pathogen to-date.
